# Differing Connectivity of Exner’s Area for Numbers and Letters

**DOI:** 10.3389/fnhum.2016.00281

**Published:** 2016-06-16

**Authors:** Elise Klein, Klaus Willmes, Stefanie Jung, Stefan Huber, Lucia W. Braga, Korbinian Moeller

**Affiliations:** ^1^Leibniz-Institut für WissensmedienTuebingen, Germany; ^2^Department of Neurology, Section Neuropsychology, University Hospital, Rheinisch-Westfälische Technische Hochschule (RWTH) Aachen UniversityTuebingen, Germany; ^3^Department of Psychology, Eberhard Karls UniversityTuebingen, Germany; ^4^SARAH HospitalBrasilia, Brazil; ^5^LEAD Graduate School, Eberhard Karls UniversityTuebingen, Germany

**Keywords:** Exner’s area, fiber tractography, atlas-based tractography, connectivity, dissociation between numbers and letters

## Abstract

There is a growing body of evidence indicating a crucial role of *Exner’s area* in (hand-) writing symbolic codes such as letters and words. However, a recent study reported a patient with a lesion affecting Broca’s and Exner’s area, who suffered from severe peripheral agraphia for letters but not for Arabic digits. The authors suggested a speculative account postulating differential connectivity of Exner’s area for numbers and letters in order to explain this dissociation. In the present study, we evaluated this account, employing atlas-based tractography for the patient’s anatomy, deterministic fiber-tracking as well as an automated toolkit to investigate the connectivity of Exner’s area in healthy adults. In particular, fiber pathways connecting Exner’s area with areas associated with language processing (e.g., the arcuate fascicle, ventral pathways encompassing the external/extreme capsule system) reached the inferior part of Exner’s area, while fronto-parietal fibers (e.g., the superior longitudinal fascicle) connected the upper part of Exner’s area with the intraparietal sulcus typically involved in number processing. Our results substantiated the differential connectivity account for Exner’s area by identifying the neural connections between fiber tracts and cortex areas of interest. Our data strongly suggest that white matter connectivity should be taken into account when investigating the neural underpinnings of impaired and intact human cognition.

## Introduction

Is there a difference between handwriting a letter or a single-digit number? Consider a person who is able to read aloud both, words (“mountain”) and numbers (“352”), to copy them, and to type them to dictation into a computer keyboard. Could one nevertheless expect a difference between writing an “R” or a “4” by hand?

Indeed, such a specific dissociation was recently described by Jung et al. (2015). In their single case study, patient CU was well able to type in letters, words, and pseudo-words on a computer keyboard on dictation. Also copying of words and isolated single letters was not a problem. However, spontaneous hand-writing and writing to dictation of words and even single letters was impossible, indicative of agraphia for letters/words. However, and even more interestingly, writing down single- and multi-digit numbers to dictation was largely unaffected.

To evaluate the origin of this particular impairment, Jung et al. (2015) considered CU’s brain lesion. CU was a 52-year-old patient suffering from severe peripheral agraphia following a left hemispheric stroke. He had a single lesion in parts of Broca’s Area (Brodmann Area [BA] 44/45) extending superiorly into tissue referred to as “Exner’s area” (BA 6). Exner (1881) isolated agraphic symptoms as a distinct syndrome and postulated that lesions of the posterior part of the middle frontal gyrus (MFG) may lead to specific writing impairments.

A meta-analysis of neuroimaging studies on handwriting corroborated the idea that Exner’s area has a crucial role in handwriting (Planton et al., 2013; see also Roux et al., 2010). While the meta-analysis revealed a left-hemispheric parieto-frontal network to be involved in writing, Exner’s area in the posterior part of the MFG was the strongest and most reliable area subserving handwriting (Planton et al., 2013). From a theoretical point of view, Planton et al. (2013) argued that Exner’s area is a region connecting abstract graphemic representations with the motor execution programmes for handwriting. This is in line with findings based on clinical data reported by Binkofski and Buccino (2004), who described Exner’s area as the final pathway, in which linguistic impulses are transferred into writing programmes (i.e., grapheme formation and their temporal sequencing). Furthermore, the results of Planton et al. (2013) support an earlier interpretation by Roux et al. (2009) that Exner’s area serves as an interface between the allographic specification of the grapho-motor programmes and (abstract) orthographic representations. Thereby, Exner’s area integrates central and peripheral stages of handwriting (see also Roux et al., 2010).

Thus, a lesion in Exner’s area, as observed in CU, would be responsible for the general pattern of behavioral problems observed. If Exner’s area is crucial for connecting abstract graphemic representations with motor execution programmes of handwriting, CU should be impaired in handwriting any shapes of letters, words, etc. to dictation, while the ability to copy shapes, to read them or to type them into a computer keyboard should be preserved. But how does this fit with CU’s preserved ability to handwrite numbers to dictation? Could there be a different neural substrate for handwriting numbers?

Accordingly, Jung et al. (2015) argued that the encoding of numbers — even of single digits — may automatically activate semantic information about the numerical quantity information they denote (e.g., Dehaene and Cohen, 1997; Dehaene et al., 2003). There is accumulating evidence corroborating this view, even when numerical quantity information is irrelevant or detrimental for solving the task at hand (e.g., Eger et al., 2003; Klein et al., 2010). Thus, Jung et al. (2015) suggested that activation of this semantic meaning might have helped the patient to handwrite numbers to dictation. Nevertheless, the question remains how such an activation of semantic information might be of any help when Exner’s area is lesioned—as in CU.

Considering this fact, Jung et al. (2015) indicated that — like many other cortical areas — Exner’s area may be connected to other cortical areas via different dorsal and ventral fiber pathways. Furthermore, the authors suggested that the activation of semantic information about a number’s magnitude, which is associated with the bilateral intraparietal sulcus (IPS; Dehaene et al., 2003; Arsalidou and Taylor, 2011), might have led to additional input into preserved upper parts of Exner’s area via a dorsal connection in CU. In contrast, the specific process of phoneme-to-grapheme conversion is traditionally viewed as a dorsal pathway function. Most often it was associated with the arcuate fascicle (Kemmerer, 2014), which connects Broca’s and Wernicke’s area (Catani and ffytche, 2005). Moreover, Andrews (2016) proposed that with increasing automation of reading and writing processes the dorsal pathway may be used less. Instead, a more ventral pathway implying no sub-vocal accompagniment may be preferred (Andrews, 2016). This is in line with the fact that letters and words are typically processed in left-hemispheric language areas (for a meta-analysis cf. Vigneau et al., 2006), which should be connected to Exner’s area encompassing ventral pathways as well.

Unfortunately, a direct reconstruction of the white matter affected by CU’s lesion was impossible because we could not acquire diffusion tensor imaging data (DTI) in him. Thus, the suggestion of Jung et al. (2015) that differential connectivity of Exner’s area with brain areas associated with the processing of numbers and language remains speculative unless it is evaluated by means of fiber-tracking. Importantly, however, the hypothesis of Jung et al. (2015) should be based on general anatomical realities. The assumption should hold for healthy subjects in general, independent of age. Thus we find it reasonable to evaluate this account without DTI data acquired in CU.

In the current study, we pursued this idea by means of three different approaches, namely by: (i) atlas-based tractography for the brain imaging data of CU; (ii) fiber-tracking in a sample of young healthy adults; and (iii) using an automated toolkit to create disconnectome maps. In case the propositions of Jung et al. (2015) were correct in principle, fiber-tracking results should substantiate differing connectivity of Exner’s area for the processing of numbers and language. Thereby, the aim of the present study was to find neuro-functional and neuro-structural evidence for the dissociation observed between handwriting numbers and letters in CU.

## Materials and Methods

### Atlas-Based Tractography for CU

#### Patient

CU was a 52-year-old right-handed former civil engineer suffering from severe peripheral agraphia due to a left hemisphere stroke after spontaneous dissection of the internal carotid artery 3 years and 6 months before the study. Initially, CU also showed severe mixed transcortical aphasia and right hemiparesis. At the time of the study, reading aloud as well as composing words or phrases from cards showing graphemes or morphemes, respectively, was well preserved^1^.

#### MRI Data Acquisition

For CU, a high-resolution T1 anatomical scan was obtained (repetition time (TR) = 19 s, matrix = 256 × 256 mm^2^, 190 slices, voxel size = 1 × 1 × 1 mm^3^; echo times (TE) = 4.9 ms; flip angle = 25°) with a 3T Siemens Magnetom Trio Tim MRI system (Siemens AG; Erlangen, Germany) at the Rheinisch-Westfälische Technische Hochschule (RWTH) Aachen University Hospital. The anatomical scan was acquired at the end of experimental sessions, the results of which have been reported elsewhere (Jung et al., 2015). The patient gave his written informed consent to participate. The study was approved by the local Ethics Committee of the Medical Faculty of the RWTH Aachen University Hospital and conducted in accordance with the latest version of the Declaration of Helsinki.

#### Atlas-Based Analysis

MRI data analysis from patient CU was performed using SPM12^2^. Imaging data was normalized into standard stereotaxic Montreal Neurological Institute space (MNI, McGill University, Montreal, QC, Canada). Image data were not smoothed in the spatial or time domain. In a second step, different fiber tracts as provided in the most recent DTI atlas available (Rojkova et al., 2015) were overlayed on CU’s anatomy in MNI space using MRIcron Software^3^, to show in a standardized space which tracts were connected to lesioned or unaffected tissue, respectively.

### Fiber-Tracking in Healthy Adults

#### Participants

DTI data were collected from a sample of 20 right-handed healthy volunteers (10 females, mean age: 23 years, range 18–26 years). Participants were scanned with the approval of the local Ethics Committee of the Medical Faculty of the Eberhard Karls University in Tuebingen. All participants gave their written informed consent. All participants had no neurological or psychiatric history and were not taking any psychoactive medication.

#### DTI Data Acquisition

MRI and DTI data were acquired on a 3T Siemens TIM Trio scanner (Siemens, Erlangen, Germany) in Tuebingen. For DTI a total of 68 scans with 69 slices was acquired using a diffusion sensitive spin-echo EPI sequence with CSF-suppression [61 diffusion encoding gradient directions (b-factor = 1000 s/mm^2^) and 8 scans without diffusion weighting (b value = 0 s/mm^2^), voxel size = 2 × 2 × 2 mm^3^, matrix size = 104 × 104 pixel^2^, TR = 11.8 s, echo times (TE) = 96 ms, TI = 2.3 s]. Furthermore, an additional high-resolution T1 anatomical scan was obtained (160 slices, voxel size = 1 × 1 ×1 mm^3^, TR = 2.2 s, TE = 2.6 ms, matrix = 256 × 256 mm^2^).

For each slice, raw diffusion-weighted data was registered and corrected simultaneously for subject motion and eddy-current induced geometrical distortions using ExploreDTI v.4.8.5^4^ (see Leemans and Jones, 2009). Constraint spherical deconvolution (CSD) was performed to estimate multiple orientations in voxels containing different populations of crossing fibers (Alexander, 2006). Afterwards whole-brain deterministic tractography was employed using an interpolated streamline algorithm that propagates from voxel to voxel, following a step length of 1 mm and a maximum angle threshold of 35°. Fractional anisotropy (FA), a scalar value that captures the degree of diffusion anisotropy, was computed from the eigenvalues of the diffusion tensor along the defined segments (Basser and Pierpaoli, 1996). Voxels showing FA values below 0.2 were excluded from tractography (Jones et al., 2002; Jones, 2003, 2004). The motion-corrected whole-brain tractography was then imported to TrackVis^5^ (Wedeen et al., 2008). This fiber-tracking software allows—amongst other features—for the identification of the tracts and their visualization in 3-dimensional (3D) space.

#### Fiber-Tracking Analysis

We aimed at evaluating the connectivity of Exner’s area with the two most important activation sites reported in Jung et al. (2015), i.e. (i) intraparietal cortex, which is associated with processing numbers in general (without the need to write them down, e.g., Arsalidou and Taylor, 2011, for a meta-analysis) and (ii) the superior and middle temporal gyri, which are associated with writing down letters/words to dictation (although probably more at the input stage before the actual hand-writing is executed). Nevertheless, as we were interested in the processing pathways from more basic input- and content-related processing to actual hand-writing, these three areas were defined as seed regions. After co-registering the T1 anatomical scan to the b0 image, peak coordinates for left IPS, left STG and left MTG as well as Exner’s area were taken from Jung et al. (2015). In particular, coordinates for the left IPS were taken from Table S3 Jung et al. (2015), reflecting the specific contrast dictation vs. copying of numbers (MNI: −44, −46, 58). Coordinates for the left superior (MNI: −68, −24, 6) and left middle (MNI: −50, −38, −2) temporal gyrus were taken from Jung et al. (2015; Table 2), reflecting relatively stronger activation when writing down letters to dictation as compared to writing down the mental images of the letters. Finally, the center of the lesion within the MFG was determined (MNI: −36, 4, 54).^6^ Then all coordinates were transferred from MNI space to the native space of each participant’s DTI data, using the inverse normalization parameters obtained during segmentation of the T1 anatomical scan (unified segmentation as implemented in SPM12) and enlarged to a sphere with a radius of 4 mm to reach white matter with simultaneous avoidance of bias due to manual hand-drawing (e.g., Kreher et al., 2008; for a similar procedure in probabilistic tracking; Willmes et al., 2014; for deterministic tracking). These spheres defined the seed regions for the fiber-tracking procedure. To substantiate the tracking results, we switched seed and target regions in our analysis. This means that each seed region also served as target region and the other way around. Only fibers connecting region A to region B but also region B to region A were considered meaningful and are reported in the “Results” Section.

After having acquired whole-brain tractography for the 20 individual participants’ data sets, these tracts were spatially normalized and averaged using a method similar to the one previously described by Jones et al. (2002) and Catani and Thiebaut de Schotten (2008). The three main fiber pathways described in the results section below were identified in each of the 20 participants. Similar to Catani and Thiebaut de Schotten (2008) one representative data set was used to perform virtual dissections (Figure 2).

**Figure 1:**
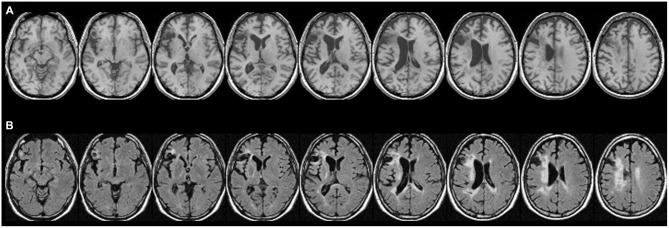
**Patient CU’s anatomy in standard Montreal Neurological Institute (MNI) space on transverse slices. (A)** Depicts a high-resolution T1-weighted structural MRI, while **(B)** shows a fluid-attenuated inversion recovery (FLAIR) scan. In most cases, T2-weighted images such as FLAIR scans show more extensive injury (e.g., gliosis) that is difficult to detect on T1-weighted scans. While large parts of Exner’s area and the ventral pathway are replaced by a pseudocyst following a colliquative necrosis, parts of the superior longitudinal fascicle (SLF II), might be affected by some gliosis (white tissue).

**Figure 2:**
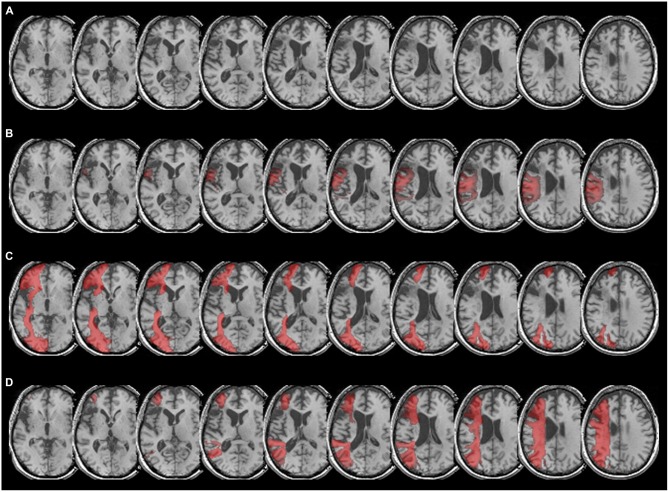
**(A)** Patient CU’s anatomy in standard MNI space on transverse slices of a high-resolution T1-weighted structural fMRI scan. The lesion affects Broca’s area and extends into Exner’s area due to a left-hemispheric MCA stroke after ACI dissection. **(B)** Overlay of CU’s brain with the arcuate fascicle, which connects Broca’s area (BA 44 and BA 45) with Wernicke’s area. As can be taken from the figure, the fibers of the arcuate fascicle overlap to a considerable extent with patient CU’s lesion, especially in the upper part of Broca’s as well as in the lower part of Exner’s area. **(C)** Overlay of CU’s anatomy with the inferior-fronto-occipital fascicle (IFOF), which in the temporal lobe encompasses ventrally the external/extreme capsule (EC/EmC) system, thereby connecting language areas ventrally with inferior frontal areas such as Broca’s area. As can be taken from the figure, the lesion in Broca’s area fully overlaps with the IFOF, so there is no ventral connection to Exner’s area via Broca’s area. Furthermore, it can be seen that the IFOF does not have contact with Exner’s area. **(D)** Overlay of patient CU’s anatomy with the SLF II. As can be seen clearly, the SLF II reaches into the upper part of Exner’s area connecting Exner’s area with parietal cortex.

### Automated Toolkit for Disconnectome Maps

Using the software BCB toolkit^7^ the localization of CU’s lesion in MNI 152 space was compared in an automated way with the connectivity pattern of 10 healthy participants provided by the toolbox. In a next step, disconnectome maps were computed allowing the identification of fibers probably disrupted by the lesion.

## Results

### Atlas-Based Results for CU

For the atlas-based results, patient CU’s anatomy was depicted in standard MNI space on transverse slices of a high-resolution T1-weighted structural fMRI scan (Figure 1A). As already outlined in Jung et al. (2015), CU’s lesion mainly affected Broca’s area but also extended into Exner’s area. For a better visualization of affected tissue, also fluid-attenuated inversion recovery (FLAIR) sequence images are provided (Figure 1B). In the (white) gliosis zones, it seems possible that additional white matter tissue was damaged. However, in gliotic regions typically only a small fraction of neurons (with their respective axons) is affected, which is then replaced by gliotic connective scar tissue. It can be seen that large parts of Exner’s area and the ventral pathway were replaced by a pseudocyst following a colliquative necrosis. On the other hand, only parts of the superior longitudinal fascicle (SLF II) might be affected by some gliosis.

According to the dual loop model of language processing in the brain (e.g., Weiller et al., 2011), Broca’s area is connected to other language areas such as Wernicke’s area (located within the left superior temporal gyrus) both dorsally (via the arcuate fascicle) and ventrally via the external/extreme (EC/EmC) system, which is encompassed by the inferior-fronto-occipital-fascile, (IFOF). Therefore, we first considered CU’s lesion (Figure 2A) in overlay with the arcuate fascicle (Figure 2B). As can be taken from Figure 2B, the arcuate fascicle overlapped to a considerable extent with CU’s lesion, especially in the upper part of Broca’s as well as in the main part of Exner’s area. Second, we evaluated the overlay of CU’s lesion with the IFOF, encompassing the EC/EmC system ventrally, thereby connecting temporal language areas ventrally with inferior frontal areas such as Broca’s area. Figure 2C illustrates that CU’s lesion in Broca’s area fully overlaps with the IFOF making any ventral connection to Exner’s area via Broca’s area unlikely. Furthermore, it can be seen that the IFOF does not reach Exner’s area, and thus does not seem to connect Exner’s area with any other cortical region in CU.

Finally, we inspected the overlay of CU’s lesion with the SLF II, which reflects a dorsal connection of frontal and parietal cortex areas including the IPS. Figure 2D depicts that the SLF II indeed reaches into the (intact) upper part of Exner’s area in CU connecting Exner’s area with parietal cortex, the IPS in particular.

### Fiber-Tracking Results

**Figure 3:**
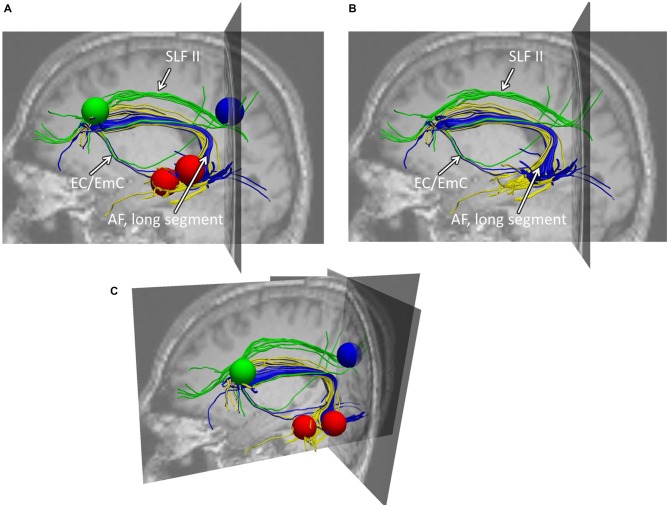
**Example of typical fiber tracking results within healthy participants. (A)** Depicts the sagittal view of both fiber trackings: (i) the connections between Exner’s area (blue sphere) to the intraparietal cortex (green sphere) via the SLF II (green fibers), (ii) the connections between Exner’s area (blue) and the superior temporal gyrus (upper red sphere) given in blue fibers (AF, longitudinal segment), and (iii) the connections between Exner’s area (blue) and the middle temporal gyrus (lower red sphere) given in yellow fibers. As can be seen, the blue fibers connect these two areas, both, dorsally (via the arcuate fascicle) as well as ventrally via the external/extreme capsule (EC/EmC) system. Importantly, both, the ventral connection as well as the dorsal arcuate fascicle reach Exner’s area considerably inferior to the SLF II. Therefore, in case the lower part of Exner’s area is affected by a lesion, there may be a dorsal fronto-parietal connection left, nevertheless. **(B,C)** Illustrate the same findings from a perspective a bit more superior and tilted towards the reader, providing also the coronal and transversal slices. **(B)** This view is given with the seed regions used, while in **(C)** only the fibers within the brain context are depicted. Note that the following (Jones et al., 2002) DTI analyses were based on data averaged across all participants, whereas segmented fibers of a representative participant are shown here for illustration purposes.

First, separate left-hemispheric trackings were run between Exner’s area and the IPS as well as Exner’s area and the superior temporal cortex (including Wernicke’s area) in all 20 participants. Interestingly, both connections were found for the fiber tracking in all 20 participants (see Figure 3). As can be taken from Figure 3, the fibers connecting Exner’s area and Wernicke’s area ran both, dorsally (via the arcuate fascicle) as well as ventrally via the external/extreme capsule (EC/EmC) system. However, both, the ventral connection as well as the dorsal arcuate fascicle reached Exner’s area considerably inferior to the SLF II (connecting Exner’s area with the intraparietal cortex). Therefore, in case the lower part of Exner’s area is affected by a lesion (as was the case in CU), there may nevertheless be a preserved dorsal fronto-parietal connection to the IPS via the SLF II.

### Automated Toolkit Results

**Figure 4:**
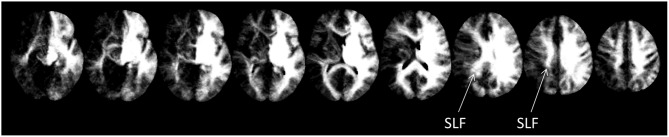
**Disconnectome maps as provided by BCBtoolkit.** The SLF II seemed to be intact, while both, the anterior segment of the AF as well as ventral fibers seemed to be affected by the lesion.

Further substantiating the results of the two previous approaches, disconnectome maps indicated that the SLF II still seemed to be intact, while both, arcuate fascicle as well as ventral fibers seemed to be disconnected by the lesion of CU. This is indicated by the missing fiber structures in Figure 4.

## Discussion

Recently, Jung et al. (2015) had reported the single case of CU, whose writing of numbers to dictation appeared largely unaffected, while spontaneous hand-writing and writing to dictation of words and even single letters was severely impaired. The present study set off to provide first neuro-structural evidence for the differing connectivity account regarding Exner’s area proposed by Jung et al. (2015) to explain this dissociation.

To this end, we employed an atlas-based analysis of the lesion data of CU, fiber-tracking in healthy adults with an intact Exner’s area, and an automated toolkit to evaluate disconnectome maps. The results of all these analyses corroborated the conclusions of Jung et al. (2015) consistently. In the tracking analysis, fiber pathways connecting Exner’s area with areas associated with language processing (e.g., the arcuate fascicle) primarily reached the lesioned inferior part of Exner’s area in CU, while fronto-parietal fibers (e.g., the SLF II) connected the upper (partly intact) part of Exner’s area with the IPS in CU. It is well possible that in healthy adults the processing of words encompasses the long segment of the arcuate fasciculus to involve Broca’s/Exner’s area, while the processing of numbers encompasses the anterior segment of the arcuate fasciculus as well. So, functions of the SLF II might be taken over by the anterior segment of the AF. This possible substitution is important for the argument, as parts of the SLF II (Figure 1B) as well as of the anterior segment of the AF (Figure 4) may be affected by gliosis in patient CU. However, at least the disconnectome maps calculated by the automated toolkit rather suggested that the SLF II should be more probably intact/less affected than the anterior segment of the AF. Taken together, all these results are well compatible with the speculative explanation of Jung et al. (2015) that (partly) intact connections with cortex areas subserving the semantic processing of number magnitude (either by the SLF II or by the anterior segment of the AF) may have led to spared handwriting of numbers in CU.

Patient CU had to handwrite either numbers or letters/words upon oral dictation. For the case of numbers there is evidence that numerical magnitude information is automatically activated when one encounters a number, which is reflected by IPS activation (e.g., Eger et al., 2003; Klein et al., 2010). This activation requires connections between temporal auditory cortex and the intraparietal cortex, probably subserved by the inferior longitudinal fascicle (ILF; e.g., Seghier, 2013). Once the IPS is involved, the semantic information may then be transferred via the SLF II to the upper part of Exner’s area (which was partially intact in CU) and may trigger the retrieval of the respective digit’s shape (see Figures 1A,B). The fiber-tracking results for healthy adults as well as the disconnectome maps further corroborate these anatomical relations: the SLF II (depicted in green in Figures 2A–C) runs much more superior and medial than the arcuate fascicle (given in yellow in Figures 2A–C) before entering into Exner’s area from superior. This substantiates the idea of a preserved dorsal connection of the upper part of Exner’s area and the IPS in CU which may account for his ability to write numbers to dictation by means of relying on number magnitude information. In line with this argument, there was strong perilesional activation observed by Jung et al. (2015) in the upper part of Exner’s area in CU when handwriting a verbally dictated number, which may reflect the retrieval of grapho-motor patterns for digits.

Yet, based on the present data from healthy adults it cannot be decided whether the semantic magnitude information of a verbally presented number might also be relayed ventrally via the EC/EmC system connecting Broca’s to Exner’s area, for instance, in case the SLF II connecting the IPS directly to Exner’s area is lesioned.

However, for letters a ventral connection encompassing the EC/EmC system and running via Broca’s area to Exner’s area situated in the direct vicinity to Area 44 seems to exist (Exner, 1881; Planton et al., 2013). Importantly, however, in the case of CU the ventral connection terminates considerably more inferior in the frontal cortex than the SLF (see also Seghier, 2013) and his lesion affected this inferior part of the frontal cortex. Therefore, numerical magnitude information from the IPS must have been transmitted most probably exclusively via the dorsal connection in CU.

This argument is further corroborated by the fiber-tracking results for the temporal language areas. Writing letters to dictation is typically associated with mostly ventral connections encompassing the EC/EmC system (Weiller et al., 2011) as well as the dorsal arcuate fascicle. Importantly, both these pathways of the dual loop model of language processing enter Exner’s area from inferior and were disrupted by CU’s lesion (Figure 1A). Therefore, it was not possible for CU to retrieve the grapho-motor patterns of letters while those for numbers may be accessible by the dorsal connections to the IPS. Of course, one might argue that the written production of numbers and words does not necessarily require a direct monosynaptic connection between posterior temporal/parietal and anterior frontal areas. The networks involved may be polysynaptic and indirect through long and short association fibers. Indeed, numerous connections to the frontal lobes have been described only recently (e.g., Catani et al., 2012). Additionally, U-fibers exist connecting Exner’s area and both inferior and superior frontal gyri. However, this does not affect our argument: the inferior part of Exner’s area of CU was lesioned and so should have been the respective U-fibers. In contrast, our data suggest the upper part of Exner’s area to be intact, so superior U-fibers might further facilitate preserved cognitive operations. Last but not least, we wish to clarify that we aimed at evaluating the account suggested by Jung et al. (2015). Future studies are needed to substantiate our results or provide evidence for another account explaining the dissociation observed in CU.

This interpretation is further substantiated by the results of a writing training, which was successfully completed by CU and reported by Jung et al. (2015). The authors trained CU to associate specific mental images with each letter (e.g., a cocktail glass for Y). After a month of intensive training, patient CU was able to write most of the letters of the alphabet. A follow-up test 6 months later revealed that he was still able to do so. Jung et al. (2015) argued that due to the training additional pictorial semantic information was attached to the letters by means of mental imagery. On the neural level mental imagery is associated with the precuneus (e.g., Ganis et al., 2004; for a review, see Cavanna and Trimble, 2006). Interestingly, there are white matter connections from the temporal cortex associated with language processing (Seghier, 2013) to the precuneus, which is directly neighboring the IPS (in fact, the sphere of 4 mm around the IPS in the fiber tracking study included parts of the precuneus as well). Thus, once the mental images associated with letters are established, their retrieval may involve the precuneus, which is connected to Exner’s area very similar to the IPS. Connections run via the SLF II to the upper part of Exner’s area, which was spared in CU. This may also explain why the mental imagery intervention was successful, allowing CU to associate grapho-motor patterns with the mental images reflecting letters.

Taken together, these results lend strong support to the hypothesis claiming differing connectivity of Exner’s area for numbers and letters. Nevertheless, it needs to be considered that our account based on fiber pathway analyses relies on fMRI data, which do not necessarily indicate that activated areas are functionally critical for the task. This uncertainty remains even though we carefully selected activations from differential contrasts as seed regions for our trackings. Therefore, further empirical evidence would be desirable whether the dissociation between letter and number writing observed in patient CU is explained sufficiently by our account of differing connectivity.

## Conclusion

Our results corroborate the conclusion of Jung et al. (2015) that the dissociation between handwriting of numbers vs. letters is due to differing connectivity of Exner’s area with cortex sites subserving number and language processing. These data indicate that domain-specificity in the human brain may evolve from the specific combination of activated cortex areas and their interconnections (see also Willmes et al., 2014). In particular, the present study indicates how informative the investigation of specific white matter connectivity can be when investigating the neural underpinnings of impaired or unimpaired human cognition.

## Author Contributions

EK, KW, SJ, and KM: participated in the idea of the manuscript. EK and SH: did the analyses. EK, KM, and KW: drafted the manuscript. LWB: provided participant data. All authors participated in the studies design, read and approved the final version of the manuscript.

## Conflict of Interest Statement

The authors declare that the research was conducted in the absence of any commercial or financial relationships that could be construed as a potential conflict of interest.

## Footnotes

^1^For a more detailed description of the neuropsychological profile of patient CU see Jung et al. (2015).

^2^http://www.fil.ion.ucl.ac.uk/spm

^3^http://www.mccauslandcenter.sc.edu/mricro/mricron/

^4^http://www.exploredti.com

^5^http://www.trackvis.org

^6^The lesion covered also the peak coordinate identified for Exner’s area in the ALE meta-analysis by Planton et al. (2013); (MNI: −22, −8, 54).

^7^www.brainconnectivitybehavior.eu
